# Assessment of Antioxidant Activity and Dose-Dependent Effect on Genotoxicity/Antigenotoxicity of *Pulmonaria officinalis* Ethanolic Extract

**DOI:** 10.3390/pharmaceutics17091134

**Published:** 2025-08-29

**Authors:** Ana Ignjatijević, Tamara Anđić, Marija Lješević, Biljana Nikolić, Tea Ganić, Stefana Spasović, Stefana Vuletić

**Affiliations:** 1 Faculty of Biology, University of Belgrade, Studentski trg 16, 11000 Belgrade, Serbia; ana.ignjatijevic@bio.bg.ac.rs (A.I.); tamara.andjic@bio.bg.ac.rs (T.A.); biljanan@bio.bg.ac.rs (B.N.); tea.ganic@bio.bg.ac.rs (T.G.); stefana.cvetkovic@bio.bg.ac.rs (S.S.); 2 Institute for Chemistry, Technology and Metallurgy, University of Belgrade, Njegoševa 12, 11001 Belgrade, Serbia; marija.ljesevic@ihtm.bg.ac.rs

**Keywords:** *Pulmonaria officinalis*, antioxidant activity, genotoxicity/antigenotoxicity, phytochemicals, dose dependent activity, hormesis

## Abstract

**Background/Objectives:** *Pulmonaria officinalis* L., commonly known as lungwort, is a medicinal plant traditionally used for respiratory ailments, but its biological activities have not yet been sufficiently researched. The aim of this study was to investigate the antioxidant and dose-dependent genotoxic/antigenotoxic properties of a 70% ethanolic extract. **Methods:** Quantification of polyphenols and GC–MS analysis were performed in order to chemically characterize the extract. Antioxidant activity was evaluated through DPPH, PFRAP, total antioxidant capacity (TAC), and ferrous ion chelating assay (FIC). MTT and alkaline comet assay were used for investigation of cytotoxicity and geno/antigenotoxicity on normal fetal fibroblast cells (MRC-5). **Results:** The chemical analysis of the extract showed that the extract is rich in polyphenolics and that phytol is the most abundant compound, accompanied by terpenoids, fatty acids, alcohols, polyketides, and alkaloids. In addition, notable antioxidant capacity was detected in all tests applied. The extract reduced cell viability only at the highest concentration tested (33.7%). Furthermore, a dual dose-dependent effect was recorded since the genotoxic effect of the tested extract was observed at higher concentrations, while non-genotoxic concentrations showed protective effects against oxidative damage of DNA. Namely, pretreatment with lungwort extract reduced the DNA damage induced by H_2_O_2_, with the highest protective effect at the lowest tested concentration, indicating a hormetic mode of action. **Conclusions:** These results provide a solid foundation for future research into this medicinal plant, with the aim of its potential therapeutic use in the prevention of diseases associated with oxidative stress.

## 1. Introduction

Among numerous definitions of oxidative stress, one of the most widely accepted and comprehensive is its description as a disparity between pro-oxidants and antioxidants in favor of the first. Reactive oxygen and nitrogen species (RONS) can be produced at elevated levels by many different mechanisms, with aerobic respiration being the main one. During oxidative phosphorylation, RONS are formed as (by-)products, primarily through electron loss from mitochondrial electron carriers and enzymes [1]. Under certain conditions, their production in living organisms can be significantly increased both endogenously (such as in a respiratory burst of polymorphonuclear leukocytes and macrophages and peroxisome stimulation) and exogenously (for example by ionizing radiation, tobacco smoke, pollutants, pesticides, and organic solvents) [2].

All RONS are harmful but some of them are more reactive, such as the hydroxyl radical, superoxide anion, and peroxynitrite, and consequently play a key role in the oxidative damage of biomolecules, including lipids, proteins, and DNA. However, it is considered that reactive oxygen species (ROS) are more abundantly distributed in the body and dominantly contribute to tissue injury [3,4]. Excessive ROS production can lead to protein oxidation, modifications of amino acids, peptide chain fragmentation, enzyme inactivation, protein denaturation, and subsequent loss of protein function [3]. Lipid peroxidation contributes to various pathological conditions by damaging cell membranes, inactivating proteins, and generating toxic by-products like malondialdehyde (MDA) and 4-hydroxy-2-nonenal (4-HNE) [3]. Additionally, as DNA is essential for cellular processes, oxidative injury to this macromolecule can have detrimental consequences. ROS-induced damage to DNA can range from minor and repairable changes to more extensive and irreversible damage, and can include a variety of lesions such as oxidation of bases and sugar–phosphate backbones, leading to apurinic/apyrimidinic (AP) site formation and single- and double-strand breaks. Moreover, ROS could contribute to formation of DNA–DNA cross-links and DNA–protein cross-links [5,6]. In addition to direct damaging effects to separate biomolecules, oxidative DNA damage can also be mediated indirectly by lipid peroxidation products [7].

On a broader scale, it has become increasingly evident that ROS are not only implicated in the progression of aging, but also play a critical role in the development and progression of various diseases, including cancer, neurodegenerative disorders, cardiovascular diseases, and muscular disorders [8,9,10,11,12,13,14,15]. Given the role of ROS in DNA damage and disease progression, the search for natural compounds with combined antioxidant and antigenotoxic properties especially against oxidative mutagens, has gained significant attention [16]. Phenolic compounds and other plant-derived metabolites play a crucial role in protecting cells from oxidative stress through various mechanisms, such as interferation with electron and/or hydrogen atom transfer [17], metal ion chelation [18], inhibition of enzymes involved in production of free radicals, regeneration of cellular antioxidants [19], and enhancement of cellular enzymatic antioxidant defense systems [17]. However, taking into account that antioxidants could act as pro-oxidants under certain conditions including application in high doses [20], attention should be paid to dosage forms used to provide better therapeutic efficacy.

*Pulmonaria officinalis* L., commonly known as lungwort, belongs to the family Boraginaceae, which includes 18 species native to Europe and western Asia [21]. Owing to its widespread distribution and use in traditional medicine, this plant is recorded in national health product databases of several countries, including Poland and Canada, highlighting its potential for pharmaceutical applications. The biological properties of *P. officinalis* could be ascribed to a diverse range of constituents belonging to different chemical groups (phenolic compounds, i.e., flavanones, flavonols, flavones, anthocyanins, lignans and polyphenolic acids, and alkaloids) [22]. Lungwort has been used for medicinal purposes since ancient times [23]. Its flowers and leaves were used as wild plant food; today, they are commonly used as ingredients in vegan and vegetarian foods, while its aerial parts are commercially available as *Pulmonariae Herba* or as ingredients in various dietary supplements, herbal blends, and teas [24]. The ethnomedicinal use of lungwort includes emollient, antitussive, expectorant, antimicrobial, diuretic, depurative, antilithiatic, and anti-inflammatory usage, as well as in respiratory and urinary disorders [22].

Taking all of the above into consideration, the aim of this study was to investigate the antioxidant capacity of the 70% ethanolic extract of *P. officinalis* and to determine its total content of phenols, flavonoids, and phenolic acids. Furthermore, the genotoxic/antigenotoxic potential of *P. officinalis* was evaluated with special emphasis on dosage forms used. In addition, the ethanolic extract was characterized by GC–MS to better assess the possible cause of bioactivity.

## 2. Materials and Methods

### 2.1. Chemicals, Reagents, and Media

Methanol and ferric chloride hexahydrate were purchased from Zorka Pharma (Šabac, Serbia). Sodium hydroxide was obtained from Centrohem (Stara Pazova, Serbia). Aluminum chloride hexahydrate, ethylenediaminetetraacetic acid (EDTA), ferrous chloride, ferrozine, potassium ferricyanide (C_6_N_6_FeK_3_), ammonium acetate (CH_3_COONH_4_), and ascorbic acid were sourced from Merck Co. (Darmstadt, Germany). Folin–Ciocalteu reagent was purchased from AppliChem-Biochemica (Darmstadt, Germany). Dulbecco’s modified Eagle’s medium (DMEM), fetal bovine serum (FBS), penicillin/streptomycin mixture, phosphate-buffered saline (PBS), trypsin from porcine pancreas, dimethyl sulfoxide (DMSO), 1% polyethylene glycol p-(1,1,3,3-tetramethylbutyl)-phenyl ether (Triton^®^ X-100), 3-(4,5-dimethylthiazol-2-yl)-2,5-diphenyltetrazolium bromide (MTT), gallic acid, quercetin, sodium carbonate, trichloroacetic acid (TCA), 2,2-diphenyl-1-picrylhydrazyl (DPPH), acridine orange, normal melting point agarose (NMP), and low melting point agarose (LMP) were purchased from Sigma-Aldrich (Steinheim, Germany). Tris(hydroxymethyl)aminomethane was obtained from SERVA (Heidelberg, Germany). Sodium dihydrogen phosphate monohydrate and disodium hydrogen phosphate dihydrate were purchased from Merck-Alkaloid (Skopje, North Macedonia). Hydrochloric acid and ammonium acetate were obtained from Fisher Scientific (Loughborough, UK).

### 2.2. Plant Material and Extraction Procedure

For purposes of this research *P. officinalis* plant material, *Pulmonariae folium*, was obtained commercially from the Institute for Medicinal Plants Research “Dr. Josif Pančić”, Belgrade, Serbia. The *P. officinalis* 70% ethanolic extract (PO) was prepared according to the method described by Cvetković et al. [25] with slight modifications. Ten grams of the plant material of lungwort were grounded and pulverized with a mortar and pestle and extracted with a 70% ethanol–water solution (1:10, *w*/*v*) at room temperature for a period of 72 h with shaking (shaker, KS 4000 i control, IKA). The extract was obtained by filtration after every 24 h using cellulose acetate syringe filters (Axiva Sichem Pvt. Ltd., Sonipat, India), followed by evaporation with a rotary evaporator (IKA RV8, IKA, Staufen, Germany) at 38 °C. The extraction yield was 9.847%. The concentrated sample was dissolved in DMSO at a concentration of 200 mg mL^−1^ and stored at −20 °C.

### 2.3. Cell Culture and Cultivation Conditions

For this study, normal fetal lung fibroblasts (MRC-5, ECACC 84101801, provided by Dr Sergej Tomić, University of Belgrade, Institute for the Application of Nuclear Energy, INEP) were used and cultured in Dulbecco’s Modified Eagle’s Medium (DMEM). The medium was enriched with 10% fetal bovine serum (FBS)*,* while a penicillin–streptomycin mixture was employed as antibiotics, in a concentration of 100 µg·mL^−1^. The cells were maintained at 37 °C in a humidified atmosphere with 5% CO_2_. Subcultivation was performed at 90% confluence with 0.1% trypsin.

### 2.4. Chemical Analysis of P. officinalis Extract

Evaporated ethanolic extract was dissolved in DMSO and analysed using a GC–MS QP2010 Ultra (Shimadzu, Kyoto, Japan) comprehensive two-dimensional gas chromatograph (GC×GC–MS). Compound separation was carried out using an RtxR-1 column (RESTEK, with CrossbondR 100% dimethyl-polysiloxane, 30 m × 0.25 mm, 0.25 μm film thickness) and a BPX50 column (SGE Analytical Science, 1 m × 0.1 mm, 0.1 μm film thickness). The oven temperature was programmed from an initial 40 °C (5 min hold) with an increase of 5 °C·min^−1^ to 300 °C, followed by a 5 min isothermal period. Modulation was not applied. Compounds were identified by spectral matching against the NIST library within a scan range of *m*/*z* 50–500.

### 2.5. Phytochemical Composition

#### 2.5.1. Total Phenolic Content (TPC)

The total phenolic content was determined using the Folin–Ciocalteu (FC) method according to Žarković et al. [25]. The tested concentration of the extract was 1 mg mL^−1^. Briefly, FC reagent was added in a volume of 500 µL to 100 µL of the extract and incubated for 6 min in the dark at room temperature. After the addition of 400 µL of 7.5% Na_2_CO_3_, the mixture was further incubated under the same conditions for 2 h. The absorbance values were measured at a wavelength of 740 nm (spectrophotometer UV-6300 PC, MRC Scientific instruments, Holon, Israel), and the TPC was calculated using the calibration curve for gallic acid (Y = 7.063X − 0.015). The results were expressed as milligrams of gallic acid equivalents (GAEs) per gram of dry weight of plant extract (dw).

#### 2.5.2. Total Flavonoid Content (TFC)

The aluminum nitrate method, previously described by [26], was used to determine TFC. The extract was tested at a concentration of 1 mg mL^−1^, while the reagent mixture contained 80% ethanol, 10% Al(NO_3_)_3_, and 1 M CH_3_COOK. After 40 min of incubation, the absorbance was measured at 415 nm against the blank. The TFC was calculated using the calibration curve for quercetin hydrate. The results were expressed as milligrams of quercetin hydrate (QHE) per gram of dry weight of plant extract (dw).

#### 2.5.3. Total Phenolic Acid Content (TPA)

To determine the TPA content in lungwort extract, the method described by Matkowski et al. was performed [27]. In the tubes, 1 mL of the extract at a concentration of 1 mg·mL^−1^, 2 mL of 0.5 M HCl, 2 mL of Arnow reagent, 2 mL of 1 M NaOH, and 3 mL of distilled water were mixed. The absorbances were measured immediately at 490 nm. The TPA content was calculated using the calibration curve for caffeic acid, and the results were expressed as milligrams of caffeic acid equivalents (CAEs) per gram of dry weight of plant extract (dw).

### 2.6. Antioxidant Activity

#### 2.6.1. DPPH Free Radical Scavenging Assay

The ability of the lungwort extract to scavenge radicals was determined using the 2,2-diphenyl-1-picrylhydrazyl (DPPH) test according to Vuletić et al. [28]. The extract was tested in a concentration range of 0.9–15.6 µg·mL^−1^. The reagent mixture containing 790 µL of methanol, 10 µL of the extract, and 200 µL of DPPH, was incubated in the dark for 30 min. The absorbance at 517 nm was measured, and the inhibition of the DPPH radical was calculated using the following equation:

(1) $$I\left(\%\right)\;=\;100\;\times \frac{A_{1\;}-{\;A}_{0}}{A_{1}}$$

where A_1_ is the absorbance of the DPPH control solution and A_0_ is the absorbance of a test sample. Ascorbic acid was used as a positive control. Furthermore, effective concentrations (EC_50_) at which DPPH radicals are scavenged by 50% were calculated.

#### 2.6.2. Ferrous Ion Chelating Capacity (FIC)

Ferrous ion chelating ability (FIC) was evaluated as described by Cvetković et al. [29]. In brief, 3.7 mL of methanol was added to 1 mL of the extract at the final concentration (1–5 mg·mL^−1^), and the mixture was incubated at 60 °C for 15 min. In addition, 100 µL of 2 mM ferrous chloride was added to allow chelation of ferrous ions. The mixture was then supplemented with 200 µL of 5 mM ferrozine to chelate the remaining unbound ferrous ions and form a purple complex. After a further 10 min of incubation, the absorbance was measured at 562 nm, and the chelating capacity was calculated using the following equation:

(2) $$\mathrm{Chelating}\;\mathrm{ability}\left(\%\right)\;=\;100\;\times \;\frac{A_{b}-A_{s}}{A_{b}}$$

where A_b_ represents absorbance of a blank and A_s_ represents absorbance of the test sample. Ethylenediaminetetraacetic acid (EDTA) was used as a positive control. Effective concentration at which ferrous ions are chelated by 50% (EC_50_) was calculated as well.

#### 2.6.3. Total Antioxidant Capacity (TAC)

The total antioxidant capacity of the extract was evaluated using the phosphomolybdenum method according to Aliyu et al. [30]. The reagent solution was made by mixing 28 mM sodium phosphate, 0.6 M sulfuric acid, and 4 mM ammonium molybdate in a 1:1:1 ratio. The extract sample at a concentration of 1 mg mL^−1^ was mixed with the reagent solution in a 1:10 ratio (0.3 mL and 3 mL, respectively). After an incubation period of 90 min at 95 °C, the tubes were allowed to cool, and absorbance was measured at 695 nm against a blank. Ascorbic acid was used to establish a calibration curve (Y = 3.3758 X + 0.022), and the results were expressed as milligrams of ascorbic acid equivalents (AAEs) per gram of dry extract (dw).

#### 2.6.4. Potassium Ferricyanide Reducing Power Assay (PFRAP)

For the evaluation of the iron(III)-reducing ability of the lungwort extract, the PFRAP method was used as in Xiao et al. [31] with some modifications, exactly as in our previous work [29]. Tubes containing 1 mL of the extract (0.0156–0.25 mg·mL^−1^), 500 µL of phosphate buffer, and 500 µL of 1% potassium ferricyanide were incubated at 50 °C for 20 min. Then, 500 µL of 10% trichloroacetic acid (TCA), 2 mL of distilled water, and 400 µL of 0.1% ferric chloride were added, and the absorbance was measured at 700 nm against the blank sample. The positive control was ascorbic acid. The results are presented graphically as a ratio of the absorbance values and concentrations of the tested substances.

### 2.7. Antigenotoxicity Testing

#### 2.7.1. Determination of the Non-Cytotoxic Concentrations of *P. officinalis* Extract

To evaluate the genoprotective effect of the lungwort extract, it was essential to determine the non-cytotoxic and non-genotoxic concentrations of the *P. officinalis* extract. Cytotoxicity was investigated on a cell line of normal fetal fibroblasts (MRC-5) using the 3-(4,5-dimethylthiazol-2-yl)-2,5-diphenyltetrazolium bromide (MTT) assay as described by Cvetković et al. [29]. In a 96-well plate, 2 × 10^4^ cells were seeded and incubated at 37 °C with 5% CO_2_. The next day, cells were treated with serial dilutions of the extract in a concentration range of 0.125 to 2 mg·mL^−1^. After a further 24 h incubation under the same conditions, the MTT dye (0.5 g mL^−1^) was added. The plates were incubated for 3 h to allow the mitochondrial enzymes of the viable cells to reduce MTT to purple formazan crystals, which were then dissolved in DMSO. A microplate reader (MultiskanFC, Thermo Scientific, Shanghai, China) was used to measure the absorbance of each well at the wavelength of 570 nm. According to the following formula, cell survival (S) was calculated:

(3) $$S\left(\%\right)\;=\;100\;\times \frac{{\mathrm{OD}}_{570}\;\mathrm{test}\;\mathrm{sample}}{{\mathrm{OD}}_{570}\;\mathrm{solvent}\;\mathrm{control}}$$

The non-cytotoxic concentrations were considered to be those at which cell viability was at least 80% [29].

#### 2.7.2. Determination of the Non-Genotoxic Concentrations of *P. officinalis* Extract

Once the non-cytotoxic concentrations were determined, the comet assay according to Cvetković et al. was performed using the same cell line to determine the non-genotoxic concentrations [32]. The cells were seeded in 12-well plates with a density of 3 × 10^5^ cells per well and incubated at 37 °C with 5% CO_2_. The next day, treatments in the concentration range of 0.0156 to 0.125 mg mL^−1^ were applied, and the cells were further incubated for 24 h. Slides were prepared beforehand by covering the top with a layer of 1% normal-melting agarose (NMP), and an additional layer of 1% NMP was applied the next day. On the third day of the experiment, the cells were washed with 1 × phosphate buffered saline (PBS) and then trypsinized. After centrifugation, 30 µL of the cell suspension was mixed with 70 µL of 1% low-melting agarose (LMA) and applied to the previously prepared slides. After preparation, the slides were incubated for 1 h at 4 °C in a freshly prepared lysis buffer (2.5 M NaCl, 0.1 M EDTA, 0.01 M Tris, Triton X-100, pH 10). After lysis, the slides were transferred to an electrophoresis buffer (300 mM NaOH, 1 mM EDTA, pH 13) for 20 min to allow denaturation. Immediately afterwards, electrophoresis was carried out at 0.75 V·cm^−1^ for a further 20 min. Finally, the slides were neutralized in pH 7.5 buffer (0.4 M Tris) for 15 min at 4 °C. Acridine orange (2 μg·mL^−1^) at a volume of 20 μL per slide was used for staining, and the slides were examined with a fluorescence microscope (Leica, DMLS, Vienna, Austria, at 400× magnification, excitation filter 510–560 nm, blocking filter 590 nm). Comet IV computer software (version, Perceptive Instruments, Bury St. Edmunds, UK) was used for visualization and quantification of comets. Hydrogen peroxide (H_2_O_2_) at a concentration of 0.5 mM was used as a positive control. One hundred randomly selected nuclei per slide were analyzed in two individual experiments. The percentage of DNA in the tail of the comets, i.e., the tail intensity (TI), was used to measure the degree of DNA damage.

#### 2.7.3. Antigenotoxicity Testing

To evaluate the protective effect of lungwort extract against H_2_O_2_-induced DNA damage, the extract pretreatment procedure was applied as described by Cvetkovic et al. [29]. Cells were seeded in 12-well plates at a density of 3 × 10^5^ cells per well and incubated at 37 °C with 5% CO_2_. After 24 h of incubation and formation of a monolayer, the cells were treated with non-genotoxic concentrations of lungwort extract (3.9–15.6 µg·mL^−1^) and incubated for a further 24 h to pretreat cells with test samples. On the third day of the experiment, the medium containing extract was removed, and a new medium with 0.5 mM H_2_O_2_ was added and incubated at 4 °C for 15 min. The cells were then washed with 1× PBS, trypsinized, and subjected to the alkaline comet assay as previously described in Section 2.7.2. The genoprotective effect of the *P. officinalis* extract was shown as the percentage of damage inhibition, which was calculated using the following equation:

(4) $$I\;\left(\%\right)\;=\left(1-\;\frac{{\mathrm{TI}}_{e}}{{\mathrm{TI}}_{c}}\right)\;\times \;100\%$$

where TI_e_ represents the tail intensity of cells treated with the test sample, while TI_c_ represents the tail intensity of cells treated with the positive control, i.e., 0.5 mM hydrogen peroxide.

### 2.8. Statistical Analysis

The results of the phytochemical composition and antioxidant tests were calculated using MS Office Excel 2016. Statistical analysis of the results was performed using GraphPad Prism 8.0 software (GraphPad Software, Inc., San Diego, CA, USA). Data were analyzed using one-way analysis of variance (one-way ANOVA) followed by Dunnett’s or Tukey’s post hoc test. A significance level of *p* < 0.05 was considered statistically significant. In the case of the alkaline comet assay, the analysis was performed using Statistica 7.0 software (StatSoft, Inc., Tulsa, OK, USA). A non-parametric Mann–Whitney *U* test was used for the analysis, and the thresholds for statistical significance were set at *p* < 0.05, *p* < 0.01, and *p* < 0.001.

## 3. Results

### 3.1. Chemical Analysis of P. officinalis Extract

The obtained results of GC–MS analysis of *P. officinalis* extract are presented in Table 1. They revealed that the most abundant component in the extract was phytol, followed by numerous aliphatic alcohols (e.g., 2-hexadecanol, 1-decanol, and 1-heptatriacotanol), fatty acid esters (e.g., ethyl palmitate and glycerol esters of linolenic acid), aldehydes (e.g., 8-octadecenal), and terpenoid or spirocyclic structures such as phorbol and oxaspiro derivatives.

### 3.2. Yield of the Main Phytochemical Groups

The results of the total phenolic, flavonoid, and phenolic acid contents of the lungwort 70% ethanolic extract are presented in Table 2. The analyzed extract showed a high polyphenol content, especially of phenolic acids.

### 3.3. Antioxidant Activity of P. officinalis Extract

**Figure 1:**
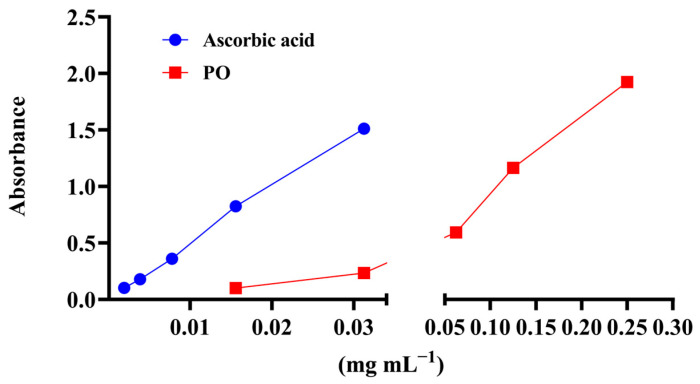
Ferric reducing antioxidant power of PO (PFRAP) assay. Results are expressed as mean value ± standard deviation of the absorbance measured at 700 nm. Ascorbic acid was used as the positive control.

The results of antioxidant activity of PO, determined in four in vitro antioxidant assays, are presented in Table 3 and Figure 1. As could be seen, the extract showed notable antioxidant activity, including a moderate ability to scavenge DPPH radicals (EC_50_ = 0.88 mg·mL^−1^), an effective ability to chelate ferrous ions (EC_50_ = 1.24 mg·mL^−1^), a considerable total antioxidant capacity (273.97 mg·AAE·gdw^−1^), as shown in Table 3, and the remarkable reducing antioxidant power of ferric iron, as determined by the PFRAP assay (absorbance of 1.924 obtained at 0.25 mg·mL^−1^, Figure 1).

### 3.4. Antigenotoxic Activity

**Figure 2:**
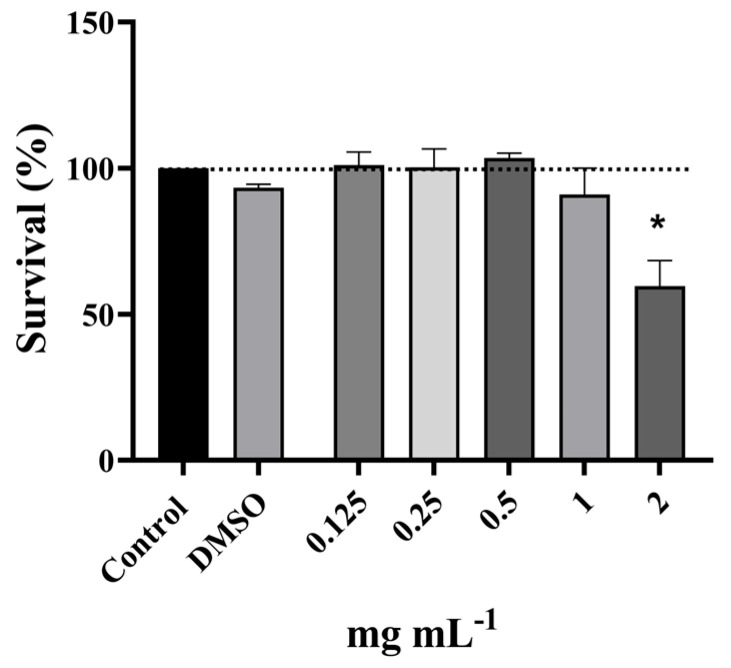
Cytotoxic potential of PO. Statistical significance in regard to solvent control (DMSO) was tested using one-way ANOVA with Dunnett’s post hoc test (* *p* < 0.05).

**Figure 3:**
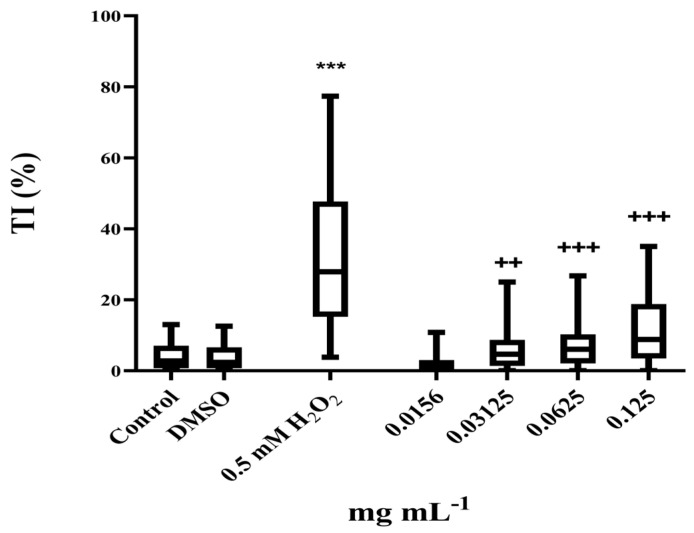
Genotoxic effect of PO. Results are presented as tail intensity (TI) percentage. Statistical significance in regard to untreated control determined for H_2_O_2_ (*** *p* < 0.001) and statistical significance in regard to DMSO determined for PO (^++^ *p* < 0.01, and ^+++^ *p* < 0.001) were tested using the nonparametric Mann–Whitney *U* test.

A preliminary evaluation of cytotoxicity on MRC-5 cells using the MTT assay revealed non-cytotoxic concentrations of the PO in the applied concentration range (0.125 mg·mL^−1^ to 2 mg·mL^−1^, Figure 2), except at the highest tested concentration (59.6%). The non-cytotoxic concentrations were used in genotoxicity testing to determine the doses that were also non-genotoxic. Results obtained showed that the concentrations in the range 0.25–1 mg mL^−1^ were extremely genotoxic, inducing complete nucleoid destruction and consequently an unmeasurable score, while the lower concentration range (0.03–0.125 mg·mL^−1^) also induced measurable genotoxicity (Figure 3). As presented, the highest non-genotoxic concentration that could be used in the further genoprotective experiments was 0.0156 mg·mL^−1^.

**Figure 4:**
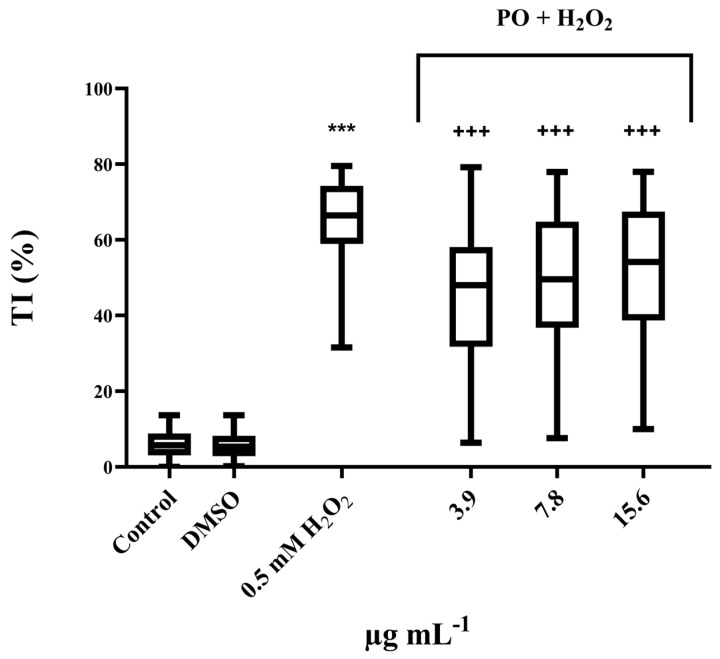
Antigenotoxic effect of PO. Results are presented as tail intensity (TI) percentage. Statistical significance in regard to untreated control determined for H_2_O_2_ (*** *p* < 0.001) and statistical significance in regard to H_2_O_2_ determined for PO (^+++^ *p* < 0.001) were tested using the nonparametric Mann–Whitney *U* test.

The protective potential of the PO extract was evaluated against H_2_O_2_–induced damage. The results presented in Figure 4 and Table 4 show that PO has an antigenotoxic effect at all tested concentrations. The highest protective effect was observed at the lowest concentration tested, with inhibition of DNA damage by 28%.

## 4. Discussion

Today, herbal products symbolize safety over synthetic products, as they are considered less toxic compared to the latter. Although herbs have been valued for centuries for their medicinal, flavorful, and aromatic qualities, the synthetic products of modern times have surpassed their importance for some time. However, the blind dependence on synthetic products is over, and people are returning to natural products with the hope of safety and security [33]. As has already been stressed, *Pulmonaria officinalis* L. (lungwort), which belongs to the Boraginaceae family, has long been used in folk medicine in many countries as a remedy for various diseases and ailments [34]. Following this global trend, the present study focuses on the evaluation of the antioxidant and genotoxic/antigenotoxic potential against oxidative DNA damage of *P. officinalis* 70% ethanol extract. As far as we know, this is the first report on the dual genotoxic/antigenotoxic effect of lungwort extract. In addition, chemical characterization of the extract has been provided as well.

It is well known that *P. officinalis* has a diverse chemical composition containing phenolic acids (gallic, rosmarinic, chlorogenic, etc.), flavonoids (rutin, luteolin, quercetin, etc.), as well as esters of caffeic acid [22]. Chemical analysis of the extract of *P. officinalis* analyzed by GC–MS in the current study revealed a complex mixture of compounds, including phytol, aliphatic alcohols (e.g., 2-hexadecanol, 1-decanol, and 1-heptatriacotanol), fatty acid esters (e.g., ethyl palmitate and glycerol esters of linolenic acid), aldehydes (e.g., 8-octadecenal), and terpenoid or spirocyclic structures such as phorbol and oxaspiro derivatives. Further, observing the obtained TPC values, it is evident that the PO extract has a really high content of phenols (286.23 mg·GAE·gdw^−1^), especially when compared to the results previously shown in the other studies of lungwort extracts [23,35]. Indeed, Neagu et al. [23] and Ivanova et al. [35] reported TPC values for lungwort extracts of 576.62 ± 6.32 × 10^−3^ mg GAE mL^−1^ and 673.39 ± 9.92 µM QE, respectively, which are both several times lower than the value obtained in this report. Furthermore, both the total flavonoid content (158.31 mg·QHE·gdw^−1^) and the total phenolic acid content (365.01 mg·CAE·gdw^−1^) further support the phytochemical richness of the extract. Therefore, such differences between our results and those reported in other studies could be related to differences in plant material or extraction conditions (type of solvent, pH, or temperature) [36]. In addition, even among plants belonging to the same species, content of secondary metabolites could be variable, depending on various external factors (climate, cultivation, and soil characteristics) and intrinsic genetic determinants, which can affect their synthesis, distribution, and storage within plant organisms. In general, different stressors elevate plants’ secondary metabolite content [37].

Considering the phytochemical composition of our extract, the issue of oxidative damage, and the well-documented strong antioxidant properties of phenolic compounds, the following part of our research was directed toward evaluating the antioxidant capacity of PO extract.

As previously mentioned RONS, such as superoxide, hydroxyl, and nitric oxide radicals, can cause significant cellular damage by targeting DNA, lipids, and proteins. This oxidative stress can be mitigated through the intake of exogenous antioxidants, primarily obtained from plant-based food and medicinal plants. The main bioactive antioxidants from these sources are polyphenols, carotenoids, and vitamins C and E [38]. Due to the diverse palette of chemical constituents, with known individual and synergistic effects, medicinal plants are considered an important source of antioxidants [39]. Preliminary studies with lungwort extracts have shown a strong antioxidant potential, probably related to the high content of flavonoids and other polyphenols, which are an important group of secondary metabolites in these plants and are known for their diverse biological activities and potential health benefits, including antioxidant capacity [23]. Indeed, Malinowska [40] determined a positive correlation between FRAP values and flavonoid content for 10 plant extracts, including of *P. officinalis*, with a coefficient correlation value of 0.931 (*r* = 0.931; *p* ≤ 0.05). When it comes to the total antioxidant capacity of PO extract, it is worth mentioning that this test provides the resultant antioxidant effect of complex mixtures, obtained by synergistic and antagonistic influence of the constituents, involving both water-soluble and fat-soluble ones [30,41]. In our study, the results showed a notable TAC value of 273.97 mg AAE g^−1^ dw, which is in accordance with high flavonoid and phenolic content.

Further, PO extract has also demonstrated significant potential in the other tests used. DPPH radical scavenging assay is the most convenient and common radical removal method for evaluating the antioxidant capacity of compounds including herbal extracts, with the EC_50_ value serving as a practical parameter to evaluate their radical-scavenging potential. In our study, the lungwort extract exhibited notable radical-scavenging potential in the DPPH assay, with an EC_50_ = 0.88 ± 0.01 mg·mL^−1^, confirming antioxidant potential. In comparison, Neagu et al. [23] reported a maximum DPPH radical-scavenging activity of 75.47% at a concentration of 3 mg mL^−1^ for the ethanolic extract of *P. officinalis*. The explanation for that may lie in the fact that phytol, a diterpene alcohol identified in our study as the most abundant in the PO, has shown significant antioxidant properties in various in vitro models. It showed remarkable antiradical potential, reducing hydroxyl radical (•OH) levels by 15%, superoxide anion radical (•O_2_^−^) levels by 23%, and nitric oxide radical (•NO) levels by 38%, while showing even greater activity against carbon dioxide anion (•CO_2_^−^) and methoxy (•CH_2_OH) radicals, with inhibition rates of 56% and 50%, respectively. Moreover, phytol at 0.1 mg·mL^−1^ concentration also showed a 48% ability to scavenge DPPH radicals [42]. In addition, phenolic compounds are widely known for their antioxidant potential, which is primarily due to the presence of hydroxyl groups and aromatic structures that enable them to scavenge free radicals [43]. Therefore, the high scavenging activity of PO extract could be attributed to these compounds.

The reducing power of *P. officinalis* extract was evaluated using the PFRAP assay, which is based on the principle that the reduction of Fe^3+^ to Fe^2+^ reflects the electron-donating capacity of the antioxidants in the sample [44]. In our study, the results showed a pronounced dose-dependent antioxidant activity within the tested concentration range, which likely arises from the synergistic activity of high levels of total phenolics, flavonoids, and phenolic acids quantified in the extract. Since iron-mediated damage to biomolecules such as lipids and DNA is involved in the development of numerous diseases such as cancer and cardiovascular and neurodegenerative diseases [45], the iron chelating mechanism of the antioxidant effect of polyphenols in addition to the free radical scavenging must be fully explored in order to fully understand the antioxidant behavior of tested PO extract.

Furthermore, the observed chelating ability of the lungwort extract could be attributed to the iron-binding ability of polyphenolics [45]. The extract exhibited a notable ferrous ion chelating capacity of 78.17% at the highest tested concentration. In addition, phytol was previously shown to effectively protect 2-deoxyribose from Fe^2+^/H_2_O_2_-induced oxidative degradation in in vitro Fenton reaction assays, demonstrating its ability to inhibit the formation of hydroxyl radicals through iron chelation [46]. Taking this result into account, phytol also contributed to the activity observed in the FIC assay.

Compared to other macromolecules in the cell, DNA is a sensitive molecule that can be damaged by environmental and endogenous factors, such as ROS. Genome instability caused by DNA damage, is a well-known cause of cancer [47] and other disorders such as the continuously rising neurodegenerative ones (Alzheimer’s, Huntington’s, and Parkinson’s diseases and amyotrophic lateral sclerosis) [48]. Most of the available evidence for *Pulmonaria* species bioactivity comes from various chemical tests and a minority of studies with cellular or biomolecule models [24] with a particular lack of studies on the genoprotective effects of *P. officinalis.* In our study, we addressed this gap by investigating not only the antioxidant activity using chemical assays, but also the antigenotoxic potential of the extract against model oxidative mutagen hydrogen peroxide [49], using the human lung fibroblast cell line MRC-5, given the important role of oxidative stress in DNA damage.

Prior to the antigenotoxic study, there was a necessity to screen for the genotoxic properties of the extract in order to clearly elucidate its effect on DNA and to determine non-genotoxic concentrations that could be used for protection [29]. The evaluation of the genotoxicity of the extract of *P. officinalis* by the alkaline comet assay, which was primarily used in our study in both genotoxicity and antigenotoxicity screening, revealed that PO exhibits a genotoxic effect at the higher tested concentrations. High concentrations of secondary metabolites found in this plant could be responsible for this result. As Demma et al. [49] reported in their study on medicinal plants, flavonoids may be the best candidates among the constituents of plant extracts for causing DNA damage in high concentrations. Indeed, flavonoids could be oxidized by peroxidases, resulting in the production of phenoxyl radicals. These intermediaries are responsible for various types of biomacromolecule injuries, inducing further oxidation of GSH, NADH, unsaturated lipids, and DNA. By depleting cellular antioxidative defense, they lead to mitochondrial toxicity and further ROS formation. Therefore, they could act as pro-oxidants, providing a background for some flavonoid cytotoxicity and genotoxicity. There is evidence that some flavonoids induce DNA fragmentation and could inhibit some DNA-processing enzymes such as topoisomerases [50]. Moreover, phytol, which is also abundant in PO, has been shown to be genotoxic, and this effect may be mediated by oxidative stress [51]. It is worth noting that the genotoxicity of plant extracts is known to vary depending on the type of extract, the part of the plant, and the genotoxicity test used, as well as on concentration range tested. Furthermore, some extracts are only genotoxic in certain assays, and results may even differ between in vitro and in vivo experiments [52].

While high concentrations of these compounds can cause DNA damage, it is important to point out that low concentrations of the same compounds can act as antioxidants that protect DNA from damage caused by various oxidizing agents such as H_2_O_2_ [46]. Hydrogen peroxide produced in the normal metabolism of aerobic organisms is a relatively unreactive species, but it induces a spectrum of DNA lesions via the formation of other ROS, mainly hydroxyl radicals. The most abundant lesions are direct single-strand breaks and oxidative base modifications. Processing of lesions by repair mechanisms and occasional direct attacks to the sugar–phosphate backbone lead to a high number of single-strand DNA breaks, which can evolve into the more lethal double-strand breaks when cells are treated with H_2_O_2_ [53,54].

In our study, a moderate protective effect against H_2_O_2_-induced DNA damage was observed after pretreatment with low doses of *P. officinalis* extract, especially at the lowest tested concentration, which proved to be the most effective. These findings showing the genotoxic effect of high doses and genoprotective properties at lower tested doses could be interpreted using the concept of hormesis, where low doses of potentially damaging agents induce protective biological effects. These substances are referred to as Janus mutagens [55,56]. Similarly to our results, a study by Abraham et al. [57] revealed that the lowest tested dose of resveratrol showed the greatest reduction in genotoxicity against nitroquinoline-1-oxide (NQO) and mitomycin C (MMC).

The presence of phenols, flavonoids, and the diterpene alcohol phytol in the extract could contribute to its antigenotoxic effect, as already reported in other studies [58,59,60]. In pretreatment experiments, some flavonoids show protective effects mainly by elevating the cellular antioxidative defense (antioxidative enzymes and reduced glutathione). Moreover, pretreatment with test substances including flavonoids can induce certain metabolizing enzymes that act as metabolic chemicals or enzymatic inactivators of mutagenic agents, or prevent the activation of promutagens [61]. Further, the intracellular pool of the extract’s bioactives, formed during pretreatment, could contribute to ROS-scavenging properties and, additionally, prevent DNA oxidation.

Based on the results of this study, it is possible that the antigenotoxic effect of PO is mediated through antioxidant mechanisms, as it showed good antioxidant activity in all assays applied. Free radicals, especially the hydroxyl radical as the most reactive, can interfere with DNA at various sites, leading to base modifications, AP sites, single- and double-breaks of strands, deletions, frame shifts, and, indirectly, DNA–protein cross-links and chromosomal aberrations. The DNA-protective potential of antioxidants lies in their ability to counteract oxidative stress by blocking the propagation of radicals, neutralizing ROS, preventing free radical oxidation reactions, and, consequently, inhibiting the formation of free lipid radicals, chelating transition metals such as Fe^2+^, and inhibiting pro-oxidant enzymes [62]. Moreover, bioactive substances from PO extract, such as phytol, could act additionally by elevating the intrinsic antioxidative defense [63]. Thus, the antigenotoxic activity observed in this study could be attributed to the synergistic effect of all abovementioned antioxidant mechanisms. However, the matter of dose should be taken into consideration, since the high doses induced some genotoxicity. For that reason, careful consideration of the optimal dose forms enabling the highest therapeutic efficacy and avoiding side effects should be provided in further investigations.

## 5. Conclusions

The present study demonstrates rich phytochemical composition with a high total content of phenols, flavonoids, and phenolic acids in the 70% ethanolic extract of *P. officinalis* L. Furthermore, the extract exhibited significant antioxidant activity in vitro, which was confirmed by DPPH, PFRAP, FIC, and TAC assays. The results obtained in the comet assay on normal fetal fibroblasts pointed out dual genotoxic properties and an antigenotoxic effect against hydrogen peroxide-induced DNA damage. Such results actually indicated a hormetic mode of action, i.e., the protective effect of low doses and detrimental effect of the higher ones. Our findings provide a solid basis for further investigation of lungwort and the underlying mechanisms of its activity in order to bring about its potential use as a source of effective therapeutics for the prevention of oxidative stress-related diseases.

## Figures and Tables

**Table 1:** GC–MS analysis of PO extract.

	Compound	Rt (min)	Relative Amount (%)
1.	Phytol	47.30	9.96
2.	5,6,6-Trimethyl-5-(3-oxobut-1-enyl)-1-oxaspiro [2.5]octan-4-one	39.60	3.68
3.	Tetracyclo [6.2.2.2(4,9).0(4,10)]tetradecan-2-one, 10,12-dihydroxy-1,3,7,8-tetramethyl-	45.10	2.38
4.	1,2,3,6-Tetrahydrobenzylalcohol, acetate	12.70	2.20
5.	Phorbol	51.40	1.78
6.	Propanoic acid, 2-methyl-, (dodecahydro-6a-hydroxy-9a-methyl-3-methylene-2,9-dioxoazuleno [4,5-b]furan-6-yl)methyl ester, [3aS-(3aα,6ß,6aα,9aß,9bα)]-	42.70	1.74
7.	2-Hexadecanol	35.00	1.63
8.	8-Octadecenal	49.40	1.62
9.	Hexadecanoic acid, ethyl ester	43.10	1.46
10.	9,12,15-Octadecatrienoic acid, 2,3-dihydroxypropyl ester, (Z,Z,Z)-	46.20	1.34
11.	12-Methyl-E,E-2,13-octadecadien-1-ol	38.20	1.23
12.	4-Methoxyphenoxyformamide, *N*-methyl-*N*-[4-(1-pyrrolidinyl)-2-butynyl]-	54.50	1.15
13.	1-Decanol, 2-methyl-	20.60	1.13
14.	Undecane, 2-methyl-	21.20	1.09
15.	5,6,6-Trimethyl-5-(3-oxobut-1-enyl)-1-oxaspiro [2.5]octan-4-one	44.40	1.09
16.	1-Heptatriacotanol	48.20	1.07
17.	Diethyl phthalate	34.00	1.02
18.	Glycine, *N*-[(3α,5ß,7α,12α)-24-oxo-3,7,12-tris[(trimethylsilyl)oxy]cholan-24-yl]-, methyl ester	59.50	1.01
19.	3-(1-Cyclopentenyl)furan	23.90	1.00

Rt—retention time.

**Table 2:** Determination of total phenolic, flavonoid, and phenolic acid contents in PO.

Assay/Extract	Total Phenolics (mg·GAE·gdw^−1^)	Total Flavonoid (mg·QHE·gdw^−1^)	Total Phenolic Acid (mg CAE gdw^−1^)
PO	286.23 ± 7.18	158.31 ± 0.27	365.01 ± 2.307

**Table 3:** Antioxidant activity of PO extract.

	EC_50_ (mg·mL^−1^)		mg·AAE·gdw^−1^
Assay/Extract	DPPH	Ferrous ion Chelating Capacity	Total Antioxidant Capacity
PO	0.88 ± 0.01	1.24 ± 0.03	273.97 ± 0.83
VitC (µg mL^−1^)	1.94 ± 0.004	nt	nt
EDTA (mg mL^−1^)	nt	0.06 ± 0.01	nt

nt—not tested.

**Table 4:** Inhibition of H_2_O_2_-induced genotoxicity.

		PO	
**µg mL^−1^**	**3.9**	**7.8**	**15.6**
Inhibition of genotoxicity	28%	23%	20%

## Data Availability

The raw data supporting the conclusions of this article will be made available by the authors on request.
